# Assessment of quality of life in patients with craniopharyngioma and identification of risk factors for compromised overall wellness

**DOI:** 10.20945/2359-4292-2023-0001

**Published:** 2023-12-01

**Authors:** Ben Lin, Shiyuan Xiang, Jiajun Chen, Yu Jing, Zhao Ye, Yichao Zhang, Xiaoyun Cao, Zhiwen Yin, Nidan Qiao, Xiang Zhou

**Affiliations:** 1 Fudan University Shanghai Medical College Huashan Hospital Shanghai China Department of Neurosurgery, Huashan Hospital, Shanghai Medical College, Fudan University, Shanghai, China; 2 National Center for Neurological Disorders Shanghai China National Center for Neurological Disorders, Shanghai, China; 3 Shanghai Key Laboratory of Brain Function and Restoration and Neural Regeneration Shanghai China Shanghai Key Laboratory of Brain Function and Restoration and Neural Regeneration, Shanghai, China; 4 Neurosurgical Institute of Fudan University Shanghai China Neurosurgical Institute of Fudan University, Shanghai, China; 5 Shanghai Clinical Medical Center of Neurosurgery Shanghai China Shanghai Clinical Medical Center of Neurosurgery, Shanghai, China; 6 Huashan Hospital West Campus Department of Nursery Shanghai China Department of Nursery, Huashan Hospital West Campus, Shanghai, China; 7 Fudan University Shanghai Medical College Department of Endocrinology Shanghai China Department of Endocrinology, Huashan Hospital, Shanghai Medical College, Fudan University, Shanghai, China

**Keywords:** Craniopharyngioma, quality of life, depression, central diabetes insipidus

## Abstract

**Objective:**

Quality of Life (QoL) has been a multifactorial concerning issue in oncology. We aimed to inspect the pre-operative QoL among patients with craniopharyngioma and to explore the potential correlations between parameters of QoL and clinical indices.

**Subjects and methods:**

We enrolled a total of 109 patients with craniopharyngioma. We utilized Short Form 36 (SF-36), Symptom Check List-90, Generalized Anxiety Disorder Questionnaire scale (GAD7), Patient Health Questionnaire Depression (PHQ9) and Pittsburgh Sleep Quality Index to prospectively evaluated their QoL. Parameters of QoL along with clinical indices were compared among sub-groups divided according to Puget classification. Correlation analyses and regression analyses were performed to detect influential determinants to self-reported wellness.

**Results:**

Patients presented impaired QoL compared with general population ( *p* < 0.001), as assessed by SF-36. Correlation analyses indicated the detrimental influence resulting from central diabetes insipidus (CDI). Multivariate linear regression unveiled the adverse effect of CDI on Mental Component Summary (coefficient = −13.869, *p* = 0.007), GAD7 total score (coefficient = 2.072, *p* = 0.049) as well as PHQ9 total score (coefficient = 3.721, *p* = 0.001). Multivariate logistic regression verified CDI as a risk factor of developing depressive symptoms (OR = 6.160, *p* = 0.001).

**Conclusion:**

QoL of patients with craniopharyngioma was remarkably compromised before operation. CDI exerted detrimental influences on patients’ QoL and it might serve as a marker for early identification of patients at risk of depression.

## INTRODUCTION

Craniopharyngioma, a type of tumor of low histological malignancy, originates from embryonic malformation of the sellar or suprasellar region. The related clinical manifestations include visual impairment, headache, and hypothalamic/pituitary hormone deficiencies ( 1 ). Besides, craniopharyngiomas involving the hypothalamic area are prone to be associated with diabetes insipidus, obesity, mental disorders, and circadian rhythm disorders ( 2 ). With the advancement of treatment, mortality of these pathological conditions are decreasing and long-term survival becomes feasible ( 3 , 4 ). The overall 10-years survival rate after surgery and/or radiotherapy reaches over 90% in recent reports. However, functional deficits including hormone deficiency, mental disturbance and circadian rhythm disorders remain and continue to be detrimental for overall wellness ( 3 ) in these patients.

Health related QoL (quality of life) has been attached to great significance in the clinical practice. Plenty of studies have focused on the influences brought by craniopharyngiomas ( 1 , 3 , 5 – 18 ). However, the substantial proportion of existing studies are retrospective study, and the scales of prospective researches engaging pre-operative evaluations are relatively small ( 3 , 5 , 17 , 18 ). Lack of information on pre-operative condition and the consequent temporal shift in QoL might render obstacles in working out better clinical practice to improve the long term QoL of these patients. Therefore, it would be crucial to explore the general status of QoL before surgery and identify potential detrimental factors.

We conducted a questionnaire-based survey among pre-operative patients suffering from craniopharyngiomas in a tertiary neurosurgical center from 2019 to 2021. The primary aim was to depict the whole picture of QoL in patients with craniopharyngiomas. The secondary aim was to identify risk factors of poor QoL by correlation and regression analyses in these patients.

## SUBJECTS AND METHODS

### Participants and methods­

Consecutive patients with craniopharyngiomas, diagnosed according to pre-operative MRI (magnetic resonance imaging) and post-operative pathology, were enrolled from the pituitary center of Huashan Hospital, Fudan University from 2018 to 2020. We excluded patients according to the following criteria: 1) failure to meet the inclusion criteria, 2) refusal to participate in the study, 3) incapability to complete the QoL assessment. This study was approved by the institutional review board of Huashan Hospital (KY2015-256) and all participants gave informed consent.

### Assessments of quality of life

The evaluation of quality of life in participants was based on self-reported questionnaires collected after admission and before surgery. For kids who were affected by the disease, they would finish the questionnaire with the assistance from their parents. The questionnaires consisted of Short Form 36 (SF-36), Symptom Check List-90 (SCL90), Generalized Anxiety Disorder Questionnaire scale (GAD-7), Patient Health Questionnaire Depression (PHQ-9) and Pittsburgh Sleep Quality Index (PSQI), with detailed rules of each question sheet presented as follows.

The Chinese version of the SF-36, translated from the International Quality of Life Assessment (IQOLA) SF-36 Standard UK Version 1.0, was utilized in the current study ( 19 ). The questionnaire composed of a single item of health transition (HT) and 35 items that could further be divided into 8 subscales: 1) physical function (PF), 2) limitations due to physical health problems (role physical, RP), 3) bodily pain (BP), 4) general health (GH), 5) vitality (VT), 6) social functioning (SF), 7) limitations due to emotional health problems (role emotional, RE), and 8) mental health. The raw scores of HT and 8 subscales were then submitted to z-score transformation and the z-transformed scores of 8 subscales were further aggregated into two summary parameters: physical component summary (PCS) scores and mental component summary (MCS) scores ( 20 – 22 ). Generally, a higher z-transformed score indicated better functional status, while a lower score indicated of a poor QoL ( 22 ).

The SCL-90, a 90-item self-report symptom inventory, was also used in our study to evaluate psychological distress and symptoms of psychopathology. Items in the inventory were clustered into 9 dimensions: Somatization (SOM), Obsessive-Compulsive (OC), Interpersonal Sensitivity (IS), Depression (DEP), Anxiety (ANX), Hostility (HOS), Phobic Anxiety (PHOB), Paranoid Ideation (PAR), and Psychoticism (PSY) ( 23 ). Global Severity Index (GSI), calculated as the mean of all 90 items in SCL-90, was further converted into T-score (mean = 50, SD = 10) to measure composite psychological distress ( 24 ). In general, a higher GSI T-score suggested an increased risk of developing psychological symptoms ( 22 , 25 ).

The Generalized Anxiety Disorder Questionnaire scale (GAD-7) was applied to assess the depressive symptoms ( 26 ). GAD-7 included 7 questions and each question had a score of 0 to 3. The total score ranged from 0 to 21, and a score ≥ 5 was identified as having anxiety symptoms ( 26 , 27 ).

The Patient Health Questionnaire Depression (PHQ-9) was used to detect depressive symptoms ( 28 ). PHQ-9 included 9 questions, and each question had a score of 0 to 3. The total score ranged from 0 to 27, and a score ≥ 5 was identified as having depressive symptoms ( 27 , 28 ).

The Pittsburgh Sleep Quality Index (PSQI) was utilized to assess recent subjective sleep quality. A subject with a total score > 6 points was defined as poor sleep quality, with the higher PSQI score indicating the worse sleep quality ( 29 ).

### Norm of Chinese population

The norms of SF-36 and SCL90 in Chinese population were based on the studies conducted by Wang and cols. ( 30 ) and Jin and cols. ( 31 ) respectively.

### Measurements of clinical indices

Medical records of each patient were reviewed to collect demographic information (age, gender, education background, marriage status and disability status), anthropometric measurements, medical comorbidities (hypertension and diabetes mellitus), past medical history (disease duration, craniopharyngioma-related surgical history, radiation history and history of epilepsy or psychological disturbance), chief complaints, ophthalmological status, neuro-radiological classification, pathologic subtypes, and pituitary functions.

Anthropometric measurements included height (cm), weight (kg) and body mass index (BMI). BMI was calculated using the following formula: BMI = weight (kg)/height (m^2^). Individual Z-scores for BMI were calculated using the following formula: Z-score = ([x]−average [x]/SD.), where [x] is the actual individual BMI, average [x] is the mean BMI, and SD is the standard deviation for the mean BMI.

After inspecting the preoperative MRI or computed tomography of the sellar region in all cases, neuro-radiological classification of each participant was determined according to the Puget classification, initially developed to evaluate the extent of hypothalamic involvement in craniopharyngiomas ( 5 ). The Puget classification stratified the participants in accordance to the degree of hypothalamic involvement as follows: Grade 0, no hypothalamic involvement; Grade 1, minor hypothalamic involvement (the tumor abutting or displacing the hypothalamus); and Grade 2, major hypothalamic involvement (the hypothalamus is not identifiable). The Puget classification of each patient was determined by two experienced radiologists (Xiang Zhou and Nidan Qiao).

Pituitary function was evaluated by experienced endocrinologists pre-operatively. Hypothalamic-pituitary-adrenal (HPA) axis disturbance was defined by basal serum cortisol level < 3 mg/dL measured at 8 a.m. or peak cortisol level < 18.1 mg/dL after insulin tolerance test or ACTH stimulation test. Hypothalamic-pituitary-thyroid (HPT) axis disturbance was diagnosed based on free T4 level below the reference range combined with a low or normal thyroid-stimulating hormone (TSH). In premenopausal women, hypothalamic-pituitary-gonadal (HPG) axis disturbance was defined by oligomenorrhea or amenorrhea combined with low serum estradiol and inappropriately low or normal follicle-stimulating hormone (FSH) and luteinizing hormone (LH) levels. HPG axis disturbance in postmenopausal women was diagnosed by serum FSH and LH within premenopausal range. In men, HPG axis disturbance was defined as low serum testosterone in conjunction with low gonadotropins. Serum insulin-like growth factor 1 (IGF-1) level was measured, although insulin tolerance test was not performed routinely to detect growth hormone deficiency in this study population. Clinical presentation, urine-specific gravity, urine and serum osmolality, serum sodium level, and the need for desmopressin treatment were comprehensively evaluated for the diagnosis of central diabetes insipidus (CDI) ( 32 ). Water deprivation testing was also performed if necessary. Moreover, all patients with pituitary axis deficits were placed on appropriate hormone replacement except for growth hormone (GH) ( 33 , 34 ). The endocrinological assessment and the associated hormone replacement were initiated in the clinic, approximately 2-3 weeks before ward admission and questionnaire administration. Also, fasting blood glucose (FBG) and serum sodium levels were documented.

### Statistical analysis

The Kolmogorov-Smirnov test was used to evaluate the normality and variance uniformity of the data. Continuous variables with normal distribution were presented as means ± SD (standard deviation), while variables with a skewed distribution were expressed as median (interquartile range). Categorical data were described as frequencies along with percentages of the total group. Z-scores of BMI are presented as mean, minimum and maximum value. Participants were divided into subgroups according to different Puget's classifications. Differences in quantitative data between subgroups were tested by Student's t-test or one-way ANOVA analysis. Differences in categorical data were determined by Chi-square analysis or fisher's exact tests as appropriate. Correlations among parameters of questionnaires and clinical indices were calculated via spearman correlation analysis. Multivariate linear or logistic regression analysis was performed to identify factors influencing each QoL as appropriate. A *p* -value threshold of < 0.05 was considered statistically significant. All statistical analyses were performed using SPSS software 23.0 (SPSS Inc, USA).

## RESULTS

### Clinical indices of enrolled patients

**Figure 1:**
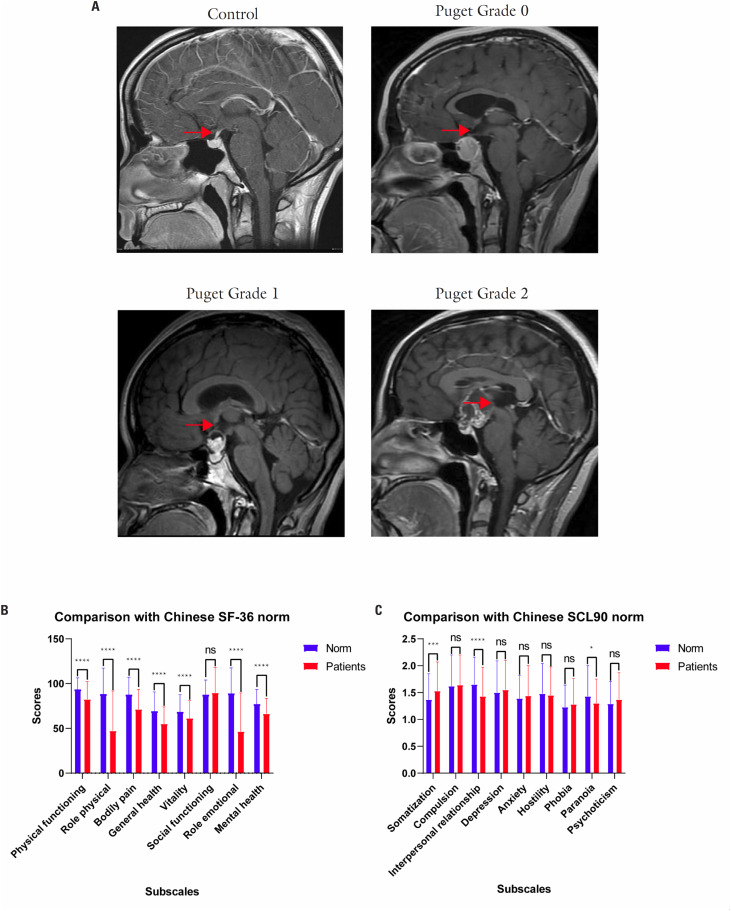
Schematic demonstration of Puget classification and divergence between patients and general population in subscales of SF36 and SCL90 ( 30 , 31 ). ( **A** ) The schematic figure demonstrated hypothalamic area in control and different extents of hypothalamic involvement in Puget classification system, with the arrows denoting the hypothalamic area. ( **B** ) Comparison of subscales in SF-36 between patients and general population illustrated a generally decreased QoL of patients. ( **C** ) Comparison of subscales in SCL90 between patients and general population demonstrated higher mean score of somatization along with lower mean scores of interpersonal relationship as well as paranoia in patients. Short Form 36 (SF-36), Symptom Check List-90 (SCL-90), quality of life (QoL). ****, *** and * denote the p value < 0.0001, p value < 0.001 and p value < 0.05.

In total, 109 patients with craniopharyngiomas (CP), confirmed by post-operative pathological examination, were enrolled in this study and the proportion of participants under 18 is 10%. Part of the patients (28, 25.7%) in this study suffered from recurrent craniopharyngioma, the surgical approaches included craniotomy (26, 23.9%) and endoscopic endonasal surgery (2, 1.8%). The patients were further stratified according to Puget classification based on pre-operative MRI, and the schematic demonstration was displayed in Figure 1A . In general, CP patients with hypothalamic involvement (P-1 & 2) presented with an increased age ( *p* < 0.001) and an elevated BMI ( *p* = 0.043) than those without hypothalamic involvement, with the remaining parameters being comparable among these groups ( Table 1 ).

**Table 1:** Clinical indices of CP patients

		Total (n = 109)	P-0 (n = 9)	P-1 (n = 55)	P-2 (n = 45)	Significance
Age (year)		40.0 (28.5-56.0)	16.0 (12.0-27.0)	47.0 (30.0-59.0)	40.0 (29.5-51.5)	<0.001
Gender						0.773
	Female	48 (44%)	5 (55.6%)	24 (43.6%)	19 (42.2%)	
	Male	61 (56%)	4 (44.4%)	31 (56.4%)	26 (57.8%)	
BMI (kg/m^2^)		24.22 ± 4.27 (0, −2.22, 2.24)	-0.79 (-2.22, 2.24)	0.06 (-2.10, 2.22)	0.09 (-1.83, 1.69)	0.043
Hypertension		18 (16.5%)	1 (11.1%)	10 (18.2%)	7 (15.6%)	0.926
Diabetes mellitus		6 (5.5%)	0 (0%)	2 (3.6%)	4 (8.9%)	0.534
Disabled		5 (4.6%)	0 (0%)	2 (3.6%)	3 (6.7%)	0.778
Disease duration (year)		10.0 (2.5-24.0)	12.0 (3.0-24.0)	6.0 (2.0-24.0)	12.0 (3.0-36.0)	0.461
**Surgical history**		28 (25.7%)	2 (22.2%)	13 (23.6%)	13 (28.9%)	0.892
	Craniotomy	26 (23.9%)	2 (22.2%)	12 (21.8%)	12 (26.7%)	
	Endoscopic endonasal surgery	2 (1.8%)	0	1 (1.8%)	1 (2.2%)	
Radiation history		5 (4.6%)	0 (0%)	2 (3.6%)	3 (6.7%)	0.778
**Chief complaint**						
	Visual deterioration	82 (75.2%)	5 (55.6%)	42 (76.4%)	35 (77.8%)	0.389
	Headache	36 (33.0%)	4 (44.4%)	15 (27.3%)	17 (37.8%)	0.414
	Polyuria	29 (26.6%)	2 (22.2%)	13 (23.6%)	14 (31.1%)	0.719
	Amenorrhea	13 (11.9%)	0 (0%)	5 (9.1%)	8 (17.8%)	0.273
	Memory disturbance	1 3(11.9%)	1 (11.1%)	3 (5.5%)	9 (20.0%)	0.059
	Ophthalmology abnormality	96 (88.1%)	6 (66.7%)	48 (87.3%)	42 (93.3%)	0.089
	Epilepsy	5 (4.6%)	0 (0%)	2 (3.6%)	3 (6.7%)	0.778
	Psychology disturbance	4 (3.7%)	0 (0%)	2 (3.6%)	2 (4.4%)	1.000
**Hormone deficiencies**						
	HPA axis	25 (22.9%)	3 (33.3%)	10 (18.2%)	12 (26.7%)	0.391
	HPT axis	84 (77.1%)	8 (88.9%)	43 (78.2%)	33 (73.3%)	0.656
	HPG axis	60 (55.0%)	6 (66.7%)	28 (50.9%)	26 (57.8%)	0.617
	IGF-1	35 (32.1%)	5 (55.6%)	13 (23.6%)	17 (37.8%)	0.105
	CDI	38 (34.9%)	3 (33.3%)	18 (32.7%)	17 (37.8%)	0.908
Number of pituitary axis deficits						0.369
	0	13 (11.9%)	1 (11.1%)	5 (9.1%)	7 (15.6%)	
	1	20 (18.3%)	1 (11.1%)	14 (25.5%)	5 (11.1%)	
	2	32 (29.4%)	1 (11.1%)	19 (34.5%)	12 (26.7%)	
	3	24 (22.0%)	3 (33.3%)	9 (16.4%)	12 (26.7%)	
	4	14 (12.8%)	2 (22.2%)	7 (12.7%)	5 (11.1%)	
	5	6 (5.5%)	1 (11.1%)	1 (1.8%)	4 (8.9%)	
FBG (mmol/L)		5.01 ± 1.24	4.61 ± 0.73	5.07 ± 1.30	5.02 ± 1.25	0.594
FT4 (pmol/L)		12.30 ± 3.51	12.19 ± 4.69	12.17 ± 3.76	12.47 ± 3.01	0.912
Na (mmol/L)		142.75 ± 3.18	142.89 ± 3.41	142.76 ± 3.23	142.71 ± 3.15	0.988

P-0: Puget grade 0; P-1: Puget grade 1; P-2: Puget grade 2; BMI: body mass index; HPA: hypothalamic-pituitary-adrenal; HPT: hypothalamic-pituitary-thyroid; HPG: hypothalamic-pituitary-gonadal; IGF-1: insulin-like growth factor 1; CDI: central diabetes insipidus; FBG: fasting blood glucose; FT4: free thyroxine; Na: sodium; continuous variables with normal distribution were presented as means ± SD (standard deviation); variables with a skewed distribution were expressed as median (interquartile range); Z-scores of BMI are presented as mean, minimum and maximum value; categorical data were described as frequencies along with percentages of the total group.

### Comparison of quality of life between patients and general population along with internal variance among patients

Compared with general population in China, mean scores of all specific dimensions of SF-36 were significantly decreased in CP ( *p* < 0.001), except that the mean score in social functioning was comparable between the two groups. In comparison to SCL90 in Chinese norm, mean score in somatization was higher ( *p* = 0.001) while mean scores in interpersonal relationship ( *p* < 0.001) as well as paranoia ( *p* = 0.020) were lower in CP ( Figures 1B and C ). And the details of comparison were provided in Supplementary Table 1 .

**Supplementary Table 1:** Comparison of parameters in SF-36 and SCL90 between patients and Chinese norm

	Patients (n = 109)	Norm	Significance
**SF-36**		**Wang and cols. (n = 3,214)**	
Physical functioning	82.43 ± 19.73	94.02 ± 12.44	<0.001
Role physical	47.25 ± 44.53	88.79 ± 28.49	<0.001
Bodily pain	71.29 ± 22.31	88.18 ± 19.02	<0.001
General health	55.07 ± 18.97	69.74 ± 20.95	<0.001
Vitality	61.33 ± 19.59	68.92 ± 18.78	<0.001
Social functioning	89.68 ± 28.35	88.03 ± 16.00	0.306
Role emotional	46.48 ± 43.04	89.57 ± 27.95	<0.001
Mental health	66.35 ± 17.22	77.61 ± 15.85	<0.001
**SCL-90**		**Jin and cols. (n = 1,388)**	
Somatization	1.53 ± 0.54	1.37 ± 0.48	0.001
Compulsion	1.64 ± 0.56	1.62 ± 0.58	0.728
Interpersonal relationship	1.43 ± 0.54	1.65 ± 0.51	<0.001
Depression	1.55 ± 0.55	1.50 ± 0.59	0.392
Anxiety	1.44 ± 0.56	1.39 ± 0.43	0.254
Hostility	1.45 ± 0.53	1.48 ± 0.56	0.589
Phobia	1.28 ± 0.48	1.23 ± 0.41	0.227
Paranoia	1.30 ± 0.45	1.43 ± 0.57	0.020
Psychoticism	1.37 ± 0.50	1.29 ± 0.42	0.059

SF-36: Short Form 36; SCL90: Symptom Check List-90; continuous variables with normal distribution were presented as means ± SD (standard deviation).

Among CP with different Puget's classifications, notably enhanced scores in interpersonal relationship ( *p* = 0.045), phobia ( *p* = 0.012) and paranoia ( *p* = 0.024) were detected in patients with no hypothalamus involvement and the remaining indexes in all questionnaires were similar ( Table 2 ).

**Table 2:** Comparison of QoL among sub-groups of patients

		P-0 (n = 9)	P-1 (n = 55)	P-2 (n = 45)	Significance
SF-36					
	Physical functioning	79.44 ± 29.10	82.91 ± 18.95	82.44 ± 18.94	0.889
	Role physical	50.00 ± 41.46	46.82 ± 45.40	47.22 ± 44.98	0.981
	Bodily pain point	76.11 ± 28.41	72.36 ± 21.59	69.02 ± 22.16	0.607
	General health	51.44 ± 15.42	55.27 ± 20.65	55.56 ± 17.72	0.836
	Vitality	63.33 ± 16.39	61.55 ± 21.56	60.67 ± 17.92	0.928
	Social functioning	88.89 ± 24.56	89.32 ± 31.95	90.28 ± 24.70	0.983
	Role emotional	33.33 (33.33-100)	66.67 (0-100)	33.33 (0-66.67)	0.178
	Mental health	61.33 ± 18.87	66.33 ± 17.97	67.38 ± 16.13	0.634
	Health transition	50.00 ± 21.65	38.64 ± 26.27	35.00 ± 25.23	0.271
	PCS	115.20 ± 29.07	114.71 ± 21.85	116.41 ± 21.20	0.930
	MCS	110.00 ± 19.33	110.79 ± 27.30	104.92 ± 24.09	0.509
SCL-90					
	Somatization	1.75 (1.33-2.04)	1.42 (1.17-1.58)	1.42 (1.08-1.75)	0.186
	Compulsion	1.80 (1.40-2.30)	1.50 (1.20-1.90)	1.50 (1.10-1.95)	0.412
	Interpersonal relationship	1.78 (1.28-2.50)	1.22 (1.11-1.44)	1.22 (1.00-1.56)	0.045
	Depression	1.85 (1.31-2.38)	1.31 (1.15-1.69)	1.38 (1.15-1.73)	0.186
	Anxiety	1.50 (1.25-2.40)	1.20 (1.00-1.70)	1.20 (1.00-1.55)	0.107
	Hostility	1.67 (1.42-2.33)	1.17 (1.00-1.67)	1.33 (1.08-1.50)	0.073
	Phobia	1.57 (1.21-2.00)	1.14 (1.00-1.43)	1.00 (1.00-1.36)	0.012
	Paranoia	1.33 (1.33-2.50)	1.17 (1.00-1.50)	1.17 (1.00-1.42)	0.024
	Psychoticism	1.60 (1.20-1.95)	1.10 (1.00-1.60)	1.20 (1.00-1.40)	0.135
	GSI	66.46 ± 5.07	63.52 ± 5.02	62.86 ± 3.09	0.080
PHQ9 total score		7.8 9± 5.23	6.65 ± 6.16	6.69 ± 5.22	0.829
Depressive symptoms					0.471
	No	2 (22.2%)	25 (45.5%)	19 (42.2%)	
	Yes	7 (77.8%)	30 (54.5%)	26 (57.8%)	
GAD7 total score		6 (2.5-7)	3 (0-8)	4 (1-8)	0.596
Anxiety symptoms					0.499
	No	3 (33.3%)	30 (54.5%)	25 (55.6%)	
	Yes	6 (66.7%)	25 (45.5%)	20 (44.4%)	
PSQI total score		7.67 ± 2.50	6.93 ± 3.70	6.38 ± 3.19	0.516
Sleep disturbance					0.348
	No	3 (33.3%)	32 (58.2%)	27 (60.0%)	
	Yes	6 (66.7%)	23 (41.8%)	18 (40.0%)	

P-0: Puget grade 0; P-1: Puget grade 1; P-2: Puget grade 2; SF-36: Short Form 36; SCL90: Symptom Check List-90; GAD-7: Generalized Anxiety Disorder Questionnaire scale; PHQ-9: Patient Health Questionnaire Depression; PSQI: Pittsburgh Sleep Quality Index; PCS: physical component summary; MCS: mental component summary; GSI: global severity; continuous variables with normal distribution were presented as means ± SD (standard deviation); variables with a skewed distribution were expressed as median (interquartile range); categorical data were described as frequencies along with percentages of the total group.

Besides, we have stratified the participants according to the number of pituitary axis deficits ( Supplementary Table 2 ), and found significant intra-subgroups difference in hostility scores ( *p* = 0.047) while the remaining parameters were comparable. Additionally, we divided the patients according to the status of HPT axis, and lower scores in physical functioning ( *p* = 0.017) and social functioning ( *p* = 0.033) were observed in patients with disturbed HPT axis, without significant alterations in other parameters of QoL ( Supplementary Table 3 ).

**Supplementary Table 2:** Comparison of QoL among patients with different number of pituitary axis deficits

		0 (n = 13)	1 (n = 20)	2 (n = 32)	3 (n = 24)	4 (n = 14)	5 (n = 6)	Significance
**SF-36**								
Physical functioning		91.15 ± 8.70	83.75 ± 16.85	83.28 ± 17.02	80.83 ± 27.33	74.64 ± 22.91	79.17 ± 12.01	0.398
Role physical		61.54 ± 42.84	46.25 ± 44.63	44.53 ± 45.67	52.08 ± 44.18	42.86 ± 47.46	25.00 ± 41.83	0.650
Bodily pain point		76.23 ± 17.43	69.80 ± 21.15	78.22 ± 22.17	67.50 ± 20.69	66.86 ± 27.40	54.17 ± 22.27	0.123
General health		57.85 ± 22.37	53.00 ± 21.37	59.19 ± 15.58	55.13 ± 17.60	50.14 ± 23.51	45.33 ± 12.03	0.480
Vitality		59.62 ± 20.36	63.25 ± 16.57	65.16 ± 17.76	60.83 ± 20.20	56.79 ± 26.21	50.83 ± 18.00	0.557
Social functioning		98.08 ± 20.31	93.13 ± 21.64	95.70 ± 28.51	84.38 ± 31.98	81.25 ± 34.23	68.75 ± 22.01	0.142
Role emotional		43.59 ± 43.85	58.33 ± 41.71	42.71 ± 41.68	51.39 ± 43.94	42.86 ± 47.91	22.22 ± 40.37	0.537
Mental health		73.23 ± 14.55	64.20 ± 18.83	68.50 ± 16.78	67.67 ± 16.68	59.71 ± 19.69	57.33 ± 10.63	0.240
Health transition		38.46 ± 16.51	32.50 ± 21.61	35.16 ± 25.29	39.58 ± 28.47	51.79 ± 31.72	33.33 ± 25.82	0.343
PCS		125.34 ± 18.99	112.89 ± 20.02	118.40 ± 21.69	114.07 ± 23.54	110.06 ± 26.51	104.89 ± 15.27	0.332
MCS		107.95 ± 25.60	113.77 ± 25.97	109.62 ± 25.60	109.67 ± 23.05	103.02 ± 30.19	90.62 ± 18.05	0.473
**SCL-90**								
Somatization		1.56 ± 0.78	1.46 ± 0.47	1.45 ± 0.43	1.54 ± 0.47	1.75 ± 0.79	1.53 ± 0.28	0.637
Compulsion		1.44 ± 0.40	1.63 ± 0.47	1.57 ± 0.44	1.71 ± 0.65	1.84 ± 0.82	1.73 ± 0.57	0.466
Interpersonal relationship		1.23 ± 0.39	1.38 ± 0.46	1.32 ± 0.37	1.45 ± 0.51	1.78 ± 0.90	1.67 ± 0.54	0.056
Depression		1.49 ± 0.51	1.44 ± 0.36	1.50 ± 0.40	1.57 ± 0.64	1.77 ± 0.88	1.74 ± 0.56	0.533
Anxiety		1.41 ± 0.68	1.3 3 ± 0.46	1.34 ± 0.34	1.50 ± 0.55	1.74 ± 0.92	1.52 ± 0.52	0.297
Hostility		1.27 ± 0.27	1.47 ± 0.46	1.38 ± 0.36	1.38 ± 0.47	1.86 ± 0.97	1.53 ± 0.50	0.047
Phobia		1.20 ± 0.39	1.16 ± 0.28	1.23 ± 0.31	1.34 ± 0.59	1.59 ± 0.76	1.24 ± 0.38	0.126
Paranoia		1.14 ± 0.20	1.27 ± 0.39	1.21 ± 0.24	1.33 ± 0.53	1.62 ± 0.77	1.44 ± 0.31	0.057
Psychoticism		1.29 ± 0.52	1.24 ± 0.36	1.28 ± 0.26	1.43 ± 0.48	1.70 ± 0.90	1.52 ± 0.45	0.088
GSI		62.60 ± 4.21	62.78 ± 3.49	62.75 ± 2.67	63.77 ± 4.48	66.09 ± 7.57	64.46 ± 3.85	0.203
PHQ9 total score		6.08 ± 7.47	5.95 ± 5.08	6.38 ± 4.39	5.88 ± 5.10	9.00 ± 7.34	11.50 ± 5.89	0.172
Depressive symptoms								0.595
	No	8 (61.5%)	10 (50.0%)	12 (37.5%)	12 (50.0%)	3 (21.4%)	1 (16.7%)	
	Yes	5 (38.5%)	10 (50.0%)	20 (62.5%)	12 (50.0%)	11 (78.6%)	5 (83.3%)	
GAD7 total score		5.08 ± 6.24	4.35 ± 4.70	4.78 ± 4.97	4.50 ± 4.63	6.43 ± 6.27	9.83 ± 6.05	0.262
Anxiety symptoms								0.757
	No	8 (61.5%)	12 (60.0%)	17 (53.1%)	14 (58.3%)	6 (42.9%)	1 (16.7%)	
	Yes	5 (38.5%)	8 (40.0%)	15 (46.9%)	10 (41.7%)	8 (57.1%)	5 (83.3%)	
PSQI total score		6.23 ± 3.75	6.65 ± 3.54	7.19 ± 2.81	6.88 ± 3.85	6.71 ± 3.65	5.67 ± 3.72	0.919
Sleep disturbance								0.829
	No	6 (46.2%)	13 (65.0%)	16 (50.0%)	13 (54.2%)	10 (71.4%)	4 (66.7%)	
	Yes	7 (53.8%)	7 (35.0%)	16 (50.0%)	11 (45.8%)	4 (28.6%)	2 (33.3%)	

SF-36: Short Form 36; SCL90: Symptom Check List-90; GAD-7: Generalized Anxiety Disorder Questionnaire scale; PHQ-9: Patient Health Questionnaire Depression; PSQI: Pittsburgh Sleep Quality Index; PCS: physical component summary; MCS: mental component summary; GSI: global severity; continuous variables with normal distribution were presented as means ± SD (standard deviation); variables with a skewed distribution were expressed as median (interquartile range); categorical data were described as frequencies along with percentages of the total group.

**Supplementary Table 3:** Comparison of QoL among patients with different status of HPT axis

		HPT normal (n = 25)	HPT disturbed (n = 84)	Significance
**SF-36**				
Physical functioning		88.40 ± 10.77	80.66 ± 21.42	0.017
Role physical		56.00 ± 45.23	44.64 ± 44.26	0.265
Bodily pain point		73.44 ± 18.57	70.66 ± 23.36	0.586
General health		56.04 ± 21.73	54.79 ± 18.21	0.773
Vitality		59.00 ± 18.43	62.02 ± 19.97	0.501
Social functioning		98.50 ± 20.51	87.05 ± 29.90	0.033
Role emotional		46.67 ± 44.10	46.43 ± 42.99	0.981
Mental health		67.20 ± 17.96	66.10 ± 17.09	0.780
Health transition		38.00 ± 17.85	38.10 ± 27.57	0.984
PCS		121.61 ± 19.08	113.61 ± 22.60	0.111
MCS		107.94 ± 25.83	108.41 ± 25.42	0.936
**SCL-90**				
Somatization		1.54 ± 0.65	1.52 ± 0.51	0.911
Compulsion		1.54 ± 0.47	1.67 ± 0.58	0.332
Interpersonal relationship		1.27 ± 0.40	1.47 ± 0.56	0.090
Depression		1.46 ± 0.46	1.58 ± 0.58	0.353
Anxiety		1.38 ± 0.60	1.46 ± 0.55	0.564
Hostility		1.34 ± 0.36	1.48 ± 0.57	0.137
Phobia		1.23 ± 0.48	1.30 ± 0.48	0.516
Paranoia		1.21 ± 0.37	1.33 ± 0.47	0.220
Psychoticism		1.27 ± 0.46	1.41 ± 0.51	0.228
GSI		62.74 ± 4.05	63.71 ± 4.49	0.337
PHQ9 total score		5.76 ± 6.02	7.07 ± 5.57	0.313
Depressive symptoms				0.112
	No	14 (56.0%)	32 (38.1%)	
	Yes	11 (44.0%)	52 (61.9%)	
GAD7 total score		4.68 ± 5.59	5.31 ± 5.23	0.604
Anxiety symptoms				0.218
	No	16 (64.0%)	42 (50.0%)	
	Yes	9 (36.0%)	42 (50.0%)	
PSQI total score		6.44 ± 3.54	6.86 ± 3.38	0.593
Sleep disturbance				0.575
	No	13 (52.0%)	49 (58.3%)	
	Yes	12 (48.0%)	35 (41.7%)	

HPT: hypothalamic-pituitary-thyroid; SF-36: Short Form 36; SCL90: Symptom Check List-90; GAD-7: Generalized Anxiety Disorder Questionnaire scale; PHQ-9: Patient Health Questionnaire Depression; PSQI: Pittsburgh Sleep Quality Index; PCS: physical component summary; MCS: mental component summary; GSI: global severity; continuous variables with normal distribution were presented as means ± SD (standard deviation); variables with a skewed distribution were expressed as median (interquartile range); categorical data were described as frequencies along with percentages of the total group.

### Correlations between quality of life and clinical indices

**Figure 2:**
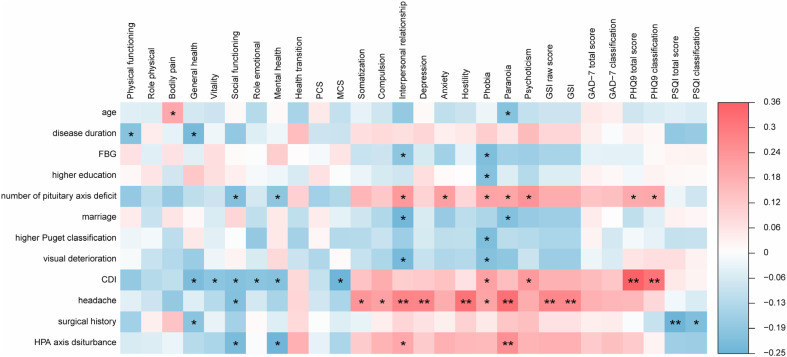
Correlation heat map between parameters of QoL and clinical indices of the patients. Correlation analysis between parameters of SF-36, SCL90, GAD7, PHQ9 along with PSQI and clinical indices. Short Form 36 (SF-36), Symptom Check List-90 (SCL90), Generalized Anxiety Disorder Questionnaire scale (GAD-7), Patient Health Questionnaire Depression (PHQ-9), Pittsburgh Sleep Quality Index (PSQI) and quality of life (QoL). The depth of color directly shows the degree of correlation between clinical indices and parameters of QoL. At the same time, correlation significance test was carried out, with * and ** symbolizing *p* < 0.05 and *p* < 0.01 respectively.

To further explore the associations between clinical indices and QoL, we conducted correlation analyses ( Figure 2 ). Regarding SF-36 questionnaire, associations in negative manner could be detected between CDI and general health (r = −0.218, *p* = 0.023), vitality (r = −0.194, *p* = 0.043), social functioning (r = −0.206, *p* = 0.032), role emotional (r = −0.196, *p* = 0.041), mental health (r = −0.204, *p* = 0.034), MCS (r = −0.245, *p* = 0.010) respectively. Additionally, we revealed that social functioning (r = −0.210, *p* = 0.028) and mental health (r = −0.189, *p* = 0.049) were negatively associated with the number of pituitary axis deficits. As for SCL-90 questionnaire, headache was remarkably associated with nearly all subscales (except for psychoticism and anxiety) and GSI in a positive manner. Similarly, interpersonal relationship (r = 0.228, *p* = 0.017), anxiety (r = 0.214, *p* = 0.025), phobia (r = 0.204, *p* = 0.033), paranoia (r = 0.200, *p* = 0.037), psychoticism (r = 0.242, *p* = 0.011) were positively correlated with the number of pituitary axis deficits. Concerning the other three questionnaires, PHQ9 total scores and PHQ9 classifications were positively associated with CDI (PHQ9 total scores: r = 0.362, *p* < 0.001; PHQ9 classification: r = 0.313, *p* = 0.001), meanwhile PHQ9 total score (r = 0.192, *p* = 0.046) and PHQ9 classification (r = 0.193, *p* = 0.045) were also positively correlated with the number of pituitary axis deficits. Turning to PSQI, the classification (r = −0.215, *p* = 0.025) and the total score (r = −0.248, *p* = 0.009) were negatively related to surgical history.

### Identifying independent risk factors for QoL

To further unveil the risk factors of impaired QoL, we conducted regression analyses. CDI (B = −13.869, *p* = 0.007) was independently associated with MCS in the multivariate linear regression model. Headache (B = 3.007, *p* < 0.001), radiation history (B = 7.925, *p* < 0.001), higher Puget classification (B = −2.054, *p* = 0.001), amenorrhea (B = 2.480, *p* = 0.035) and FBG (B = −0.683, *p* = 0.037) were demonstrated to be remarkable determinants of GSI in the multivariate regression model. Headache (B = 2.229, *p* = 0.040) along with CDI (B = 2.072, *p* = 0.049) was found to be an influential factor for GAD7. CDI (B = 3.721, *p* = 0.001) and headache (B = 2.934, *p* = 0.008) were verified as vital variates for PHQ9. Recurrence (B = −2.287, *p* = 0.004) and radiation history (B = 4.043, *p* = 0.014) were verified as vital variates for PSQI. However, no factors were independently associated with PCS in the linear regression model. Details of the identified risk factors in multivariate linear regression analyses were depicted in Table 3 .

**Table 3:** Multivariate linear regression analyses in CP patients identify influential factors associated with MCS, GSI and total scores of GAD7, PHQ9 and PSQI

		Beta	SE	95%CI	P value
MCS					
	CDI	-13.869	5.021	-23.827- −3.911	0.007
GSI					
	Headache	3.007	0.798	1.423-4.591	<0.001
	Radiation history	7.925	1.896	4.164-11.687	<0.001
	Higher Puget classification	-2.054	0.619	-3.282- −0.826	0.001
	Amenorrhea	2.480	1.162	0.175-4.786	0.035
	FBG	-0.683	0.324	-1.325- −0.041	0.037
GAD7 total score					
	Headache	2.229	1.070	0.106-4.351	0.040
	CDI	2.072	1.042	0.006-4.138	0.049
PHQ9 total score					
	CDI	3.721	1.062	1.614-5.827	0.001
	Headache	2.934	1.091	0.769-5.098	0.008
PSQI total score					
	Recurrence	-2.287	0.779	-3.833- −0.741	0.004
	Radiation history	4.043	1.621	0.829-7.258	0.014

MCS: mental component summary; GSI: global severity; GAD-7: Generalized Anxiety Disorder Questionnaire scale; PHQ-9: Patient Health Questionnaire Depression; PSQI: Pittsburgh Sleep Quality Index; CDI: central diabetes insipidus; SE: standard error; CI: confidence interval.

GAD7, PHQ9 or PSQI were further dichotomized as having/not anxiety symptoms, depressive symptoms and poor sleeping quality. In the subsequent multivariate logistics regression analysis, headache (OR = 2.395, *p* = 0.041) were demonstrated to be a significant risk factor of GAD7 classification (developing anxiety symptoms). Recurrence (OR = 0.326, *p* = 0.038) and CDI (OR = 6.160, *p* = 0.001) were identified as remarkable risk factors of PHQ9 (developing depressive symptoms). Recurrence (OR = 0.317, *p* = 0.019) was identified as the remarkable risk factor of PSQI (developing poor sleeping quality). And the detailed parameters of these risk factors were included in Table 4 .

**Table 4:** Multivariate logistic regression analysis indicated possible factors associated with anxiety symptoms, depressive symptoms and sleep disturbance

		B	SE	OR	95%CI	P value
GAD7						
	Headache	0.873	0.427	2.395	1.037-5.530	0.041
PHQ9						
	Recurrence	-1.122	0.540	0.326	0.113-0.938	0.038
	CDI	1.818	0.533	6.160	2.167-17.513	0.001
PSQI						
	Recurrence	-1.150	0.492	0.317	0.121-0.830	0.019

GAD-7: Generalized Anxiety Disorder Questionnaire scale; PHQ-9: Patient Health Questionnaire Depression; PSQI: Pittsburgh Sleep Quality Index; CDI: central diabetes insipidus; SE: standard error; OR: odds ratio; CI: confidence interval.

## DISCUSSION

In the current study, we evaluated the pre-operative QoL within patients with craniopharyngioma. As a consequence, we found that patients with craniopharyngioma presented an impaired QoL compared with general population except for lower mean scores of interpersonal relationship as well as paranoia in patients, probably resulting from lethargy and decreased reactivity subsequent to hypothalamic involvement ( 35 , 36 ). Besides, QoL of sub-groups among these participants were basically comparable. Additional correlation analyses in the entirety indicated the detrimental influence caused by CDI, headache, number of pituitary axis deficits and surgical history. Further multivariate linear regression in participants revealed the adverse effect of CDI, headache and tumor recurrence on quantitative parameters in QoL. And the multivariate logistic regression among all patients confirmed CDI as a risk factor of developing depressive symptoms.

Our study, to the best of our knowledge, illustrated the pre-operative QoL in Chinese people for the first time. Further correlation analyses and regression analyses unfolded the potential negative consequences on QoL associated with CDI, warranting prompt and effective peri-operative management of diabetes insipidus and early identification along with intervention of people prone to poor QoL.

Impaired QoL in CDI has been reported by some researchers ( 37 , 38 ). A patient's quality of life is often impeded as a result of the condition, for example from sleep being disturbed by nocturia ( 39 ). Treating CDI with desmopressin is generally safe and effective and shown to improve patients’ quality of life ( 40 ). However, the results regarding the effect of treatment on QoL in CDI are heterogenous. Patients with CDI often report quality of life issues, despite control of polyuria with desmopressin ( 41 ).

Although the efficacy of CDI as a marker for QoL remains controversial, it can serve as a surrogate marker of hypothalamic involvement. As reported, 80% to 90% of the magnocellular AVP neurons in the hypothalamus must be destroyed to produce polyuria and polydipsia ( 42 ), indicating extensive destruction of the AVP magnocellular neuron cell bodies is needed to induce DI ( 43 ). Thus, the establishment of CDI indicates the extensive destruction of the hypothalamus, providing the neuroanatomical basis for the development of depression. And the current treatment modalities can't reverse the anatomical deconstruction, despite the symptomatic alleviation. Indeed, adults with childhood-onset craniopharyngioma (COCP) with hypothalamic involvement, despite being on hormone replacement including GH, demonstrate the persistent impairment of cognitive function, psychosocial health, QoL and overall social functioning ( 10 , 44 ). Therefore, we propose that CDI might serve as a clinical marker of altered QoL in patients with craniopharyngioma.

The majority of previous researches were retrospective studies involving post-operative QoL in craniopharyngioma, and quantities as well as scales of prospective studies enrolling pre-operative assessment of QoL were relatively small ( 3 , 5 , 17 , 18 ). Compared with previous research, our study also demonstrated a notably impaired QoL before surgery ( 3 ). On the contrary, prior studies indicated that pre-operative hypothalamic involvement in CP patients exerted adverse influences on the post-operative QoL ( 1 , 5 , 8 , 10 , 12 ), while there was no significant divergence in pre-operative QoL among sub-groups of CP patients stratified according to hypothalamic involvement in our study except for higher mean scores of interpersonal relationship, phobia and paranoia in patients without hypothalamic involvement. Also, further regression analyses in CP patients yielded negative association between pre-operative hypothalamic involvement and GSI in SCL90. With regards to the reasons for this discrepancy, we proposed the following factors might explain it. First of all, the hypothalamic involvement might result in lethargy according to previous reports ( 35 , 36 ), thus causing decreased reactivity and subsequent lower scores in interpersonal relationship, phobia and paranoia as well as negative correlation with GSI. Further, the major part of the CP patients in our study had hypothalamic involvement and this imbalanced proportions may introduce bias into the evaluation of association between hypothalamic involvement and QoL. Last but not least, since the bulk of past researches focused exclusively on post-operative QoL, the reported association between pre-operative hypothalamic involvement and impaired QoL might be confounded by ensuing surgical or radiological destruction of hypothalamus and adjacent structures, and therefore masking the relationship between pre-operative hypothalamus involvement and QoL.

The limitations of our study could be summed up as follows. Due to the unequal numbers of patients with different extent of hypothalamus involvement in CP, the current study may introduce certain bias into the comparison and analyses. Also, the lack of paired longitudinal follow-up data on QoL as well as clinical indices would hinder us from exploring the temporal dynamics of QoL and its determinants. Last but not least, although very low levels of IGF-1 are strongly suggestive of GHD, normal IGF-1 concentrations do not exclude GHD at any age ( 45 ). Since GH and IGF-1 are critical regulators of structure and function within the nervous system ( 46 ), a more prevalent untreated GH deficiency in patients of this study might also account for the impaired QoL. Therefore, a larger cohort with balanced proportions of different sub-groups and paired follow-up data is essential.

In conclusion, this study demonstrated that QoL of craniopharyngioma was significantly compromised before surgery. Polyuria, surgical history, amenorrhea, headache and CDI were identified to exert detrimental influences on patients’ QoL.
